# Toward the Knowledge of the Epidemiological Impact of Acute Rheumatic Fever in Italy

**DOI:** 10.3389/fped.2021.746505

**Published:** 2021-12-15

**Authors:** Antonino Maria Quintilio Alberio, Filippo Pieroni, Alessandro Di Gangi, Susanna Cappelli, Giulia Bini, Sarah Abu-Rumeileh, Alessandro Orsini, Alice Bonuccelli, Diego Peroni, Nadia Assanta, Carla Gaggiano, Gabriele Simonini, Rita Consolini

**Affiliations:** ^1^Pediatrics Unit, Department of Clinical and Experimental Medicine, University of Pisa, Pisa, Italy; ^2^Rheumatology Unit, Meyer Children's Hospital, University of Florence, Florence, Italy; ^3^Pediatrics Unit, Section of Pediatric Neurology, Department of Clinical and Experimental Medicine, University of Pisa, Pisa, Italy; ^4^Heart Hospital-G. Monasterio Tuscany Foundation, Massa, Italy; ^5^Clinical Pediatrics, Department of Molecular Medicine and Development, University of Siena, Siena, Italy; ^6^Pediatrics Unit, Section of Clinical and Laboratory Immunology, Department of Clinical and Experimental Medicine, University of Pisa, Pisa, Italy

**Keywords:** acute rheumatic fever, epidemiology, revised Jones criteria, high-risk population, incidence

## Abstract

**Background:** To estimate the incidence of Acute Rheumatic Fever (ARF) in Tuscany, a region of Central Italy, evaluating the epidemiological impact of the new diagnostic guidelines, and to analyse our outcomes in the context of the Italian overview.

**Methods:** A multicenter and retrospective study was conducted involving children <18 years old living in Tuscany and diagnosed in the period between 2010 and 2019. Two groups were established based on the new diagnostic criteria: High-Risk (HR) group patients, *n* = 29 and Low-Risk group patients, *n* = 96.

**Results:** ARF annual incidence ranged from 0.91 to 7.33 out of 100,000 children in the analyzed period, with peak of incidence registered in 2019. The application of HR criteria led to an increase of ARF diagnosis of 30%. Among the overall cohort joint involvement was the most represented criteria (68%), followed by carditis (58%). High prevalence of subclinical carditis was observed (59%).

**Conclusions:** Tuscany should be considered an HR geographic area and HR criteria should be used for ARF diagnosis in this region.

## Introduction

Acute Rheumatic Fever (ARF) is a multisystemic autoimmune response to infection of the group A streptococcus (GAS). The incidence of ARF is low in high-income countries such as North America (1, 2) and Western Europe (3), but persists high in low-income countries (4, 5), and among marginalized sections of the society of high-income countries (6–8) and in the South Pacific (9–11), becoming an important cause of morbidity and mortality. Jones criteria for ARF diagnosis were first established in 1944, then revised in 1992 (12) and afterward in 2015 (13) by the American Heart Association (AHA), leading to a distinction between moderate to high-risk (HR) and low-risk (LR) populations (incidence cut-off <2 out 100,000 per year), to avoid underdiagnoses in LR populations and overdiagnoses in HR populations. Therefore, these new criteria incorporate three major changes: the above-mentioned risk stratification based on disease endemicity, inclusion of polyarthralgia or monoarthritis and monoarthralgia among major and minor criteria of HR population respectively, and subclinical carditis as a major manifestation in both LR and HR populations. Consequently, the knowledge of the incidence of ARF is an essential need for the application of the new diagnostic criteria in each country.

The incidence of ARF in Italy has been scarcely investigated and limited to the main regions of Northern and to a single region of Central Italy. We performed a multicenter retrospective study to describe the clinical picture of ARF and to estimate its incidence in Tuscany, a region of the Central Italy, focusing on the epidemiologic impact of the new diagnostic criteria for HR populations.

## Methods

### Study Design and Data Collection

Demographic data from the Italian National Institute for Statistics (ISTAT: national and governmental registry that collects data relating to the permanent census of the population and housing) were used to define the number of childhood population at risk of GAS infection living in Tuscany in the 2010–2019 period. The annual incidence of ARF was estimated using the number of children at risk for the respective year.

The diagnosis of ARF is based on the criteria of AHA revised in the 2015 year, which distinguishes the populations in two groups, HR and LR, respectively, and includes both major criteria (carditis clinical and/or subclinical, polyarthritis, erythema marginatum, chorea and subcutaneous nodules, as well as also monoarthritis and polyarthralgia only in HR populations) and minor criteria such as polyarthalgia, fever (≥38.5°C), erythrocyte sedimentation rate (ESR ≥60 mm/h) in LR populations, while monoarthralgia, fever (≥38°C), ESR ≥30 mm/h in HR populations, and in both populations C-reactive protein (CRP ≥3.0 mg/dl) and a prolonged PR interval for age variability.

We included all patients aged <18 years with ARF who were referred to the three main Pediatric Centers in Tuscany (Pisa, Florence, Siena) and to the Pediatric Cardiology Unit of the “Ospedale del Cuore” in Massa in a 10-year period between January 2010 and December 2019. Additionally, after critically evaluated, we included the patients from 2010 to 2015 discharged with related diagnoses such as Sydenham chorea, rheumatic carditis, post-streptococcal arthritis, undifferentiated arthritis or undefined polyarthralgia that did not meet ARF diagnosis; therefore, in order to establish the number of children that could have benefited from an ARF diagnosis, we retrospectively applied HR criteria in this group.

In all patients the previous streptococcal tonsillar infection was demonstrated by the evidence of elevated or rising ASO titer or by the throat swab or culture positive for GAS. An antistreptolysin O titer >240 Todd Units and/or an anti-Deoxyribonuclease B (Anti-DNase B) titer >640 Todd Units were considered evidence of streptococcal infection (14). All patients who did not meet the inclusion criteria or those patients with missing clinical and/or laboratory data were excluded.

Patients were screened according to the *International Classification of Diseases, Ninth Revision* (ICD-9) discharge codes either by manual record evaluation of discharge or outpatient letters. A cohort of 129 patients was enrolled (out of four were excluded). These selected patients (n=125) received an extensive diagnostic workup including demographic, clinical and laboratory data retrieved from medical records. All the children underwent a cardiologic assessment with electrocardiography (ECG) and two-dimensional color-flow Doppler echocardiography at the time of diagnosis and in follow-up. Subclinical carditis was classified as evidence of valvular involvement detected by echocardiography, in absence of auscultatory findings of valvular dysfunction on clinical examination. Patients with physiological aortic or mitral regurgitation, in absence of other symptoms and with anti-streptolysin antibody (ASO) titer or throat culture negative, were excluded. No sequelae were pointed out in ARF patients with joint involvement in follow-up.

Finally, the total cohort of patients was classified into two main groups according to the AHA revised ARF criteria. HR group was composed of children with confirmed ARF according to HR criteria (post-2015 HR) and of retrospectively revised patients from 2010 through June 2015 without achieved ARF diagnosis, by applying HR criteria (pre-2015 HR). LR group included children with confirmed ARF before 2015 (pre-2015 LR) and patients that would have received the diagnosis of ARF after 2015 even if LR population criteria were applied (post-2015 LR).

### Statistical Analysis

Normal distribution of the data was tested by using the Shapiro-Wilk, Skewness, Kurtosis tests. Equal variances assumptions were tested according to the modified Levene method. Parametric tests (Student *t*-test), non-parametric (Mann-Whitney, Wilcoxon rank-sum), Chi-squared or Fisher tests were used to compare age, sex, and clinical characteristics among LR and HR patients where appropriate. Statistical analysis was conducted using NCSS 2020 Statistical Software (2020). NCSS, LLC. Kaysville, Utah, USA, ncss.com/software/ncss and Microsoft Excel 365 software; *P* < 0.05 were considered statistically significant.

## Results

The childhood population (2–18 years) at risk of GAS infection was 3,300,928 children distributed from 2010 to 2019, as calculated from the data provided by ISTAT. A total of 125 patients was included. Among them, 96 patients were assigned to LR group (pre-2015 *n* = 35, post-2015 *n* = 61), while 29 children were assigned to HR group (pre-2015 *n* = 8, post-2015 *n* = 21). The main characteristics of the cohort are reported in Table 1. Age distribution showed a higher prevalence in children aged between 5 and 10 years and a mild preponderance in male patients (1.4:1). No significant differences were observed for age (HR median age = 9.5 95%CI = 7.4–10.2, LR median age = 9.4 95%CI = 8.1–9.7, *P* = 0.91) and gender (Chi squared with Yates'correction χ^2^ 0.001, *P* = 0.98) between group HR and LR patients (Table 1).

**Table 1:** Main characteristics of LR and HR patients.

	**LR Group**	**HR Group**	**Total**
Number of patients, *n* (%)	96 (77)	29 (23)	125 (100)
Median age, years (IQR)	9.36 (3.96)	9.52 (3.89)	9.41 (3.65)
Sex, n (%)	Male 56 (58)	Male 17 (59)	Male 73 (58)
	Female 40 (42)	Female 12 (41)	Female 52 (42)
Children aged <5 years, *n* (%)	5 (5)	1 (3)	6 (5)
Children aged from 5 to 10 years, *n* (%)	60 (63)	17 (59)	77 (62)
Children aged from 10 to 15 years, *n* (%)	27 (28)	10 (35)	37 (29)
Children ≥ than 15 years, *n* (%)	4 (4)	1 (3)	5 (4)
ASO titer, median (IQR)	1,375 (1,045)	1,270 (896)	1,360 (981)
Anti-DNase B, median (IQR)	926 (1,216)	1,384 (1,468)	1,109 (1,449)
Number of days between GAS infection and ARF onset, median (IQR)	11 (17)	13 (20)	12 (17)

*IQR, interquartile range; ASO, anti-streptolysin antibody; Anti-DNase B, Anti-Deoxyribonuclease B; GAS, Group A Streptococcus; ARF, acute rheumatic fever*.

The median range time between the streptococcal infection and the onset of ARF symptoms was 12 days (IQR 17). ASO and Anti-DNase B titres of both groups did not differ significantly (HR median ASO = 1,270 95%CI = 805–1,610, LR median ASO = 1,378 95%CI = 1130–1563, *P* = 0.58; HR median anti-DNase B = 1,385 95%CI = 738–2200, LR median anti-DNase B = 927 95%CI = 730–1218, *P* = 0.24) (Table 1).

**Figure 1:**
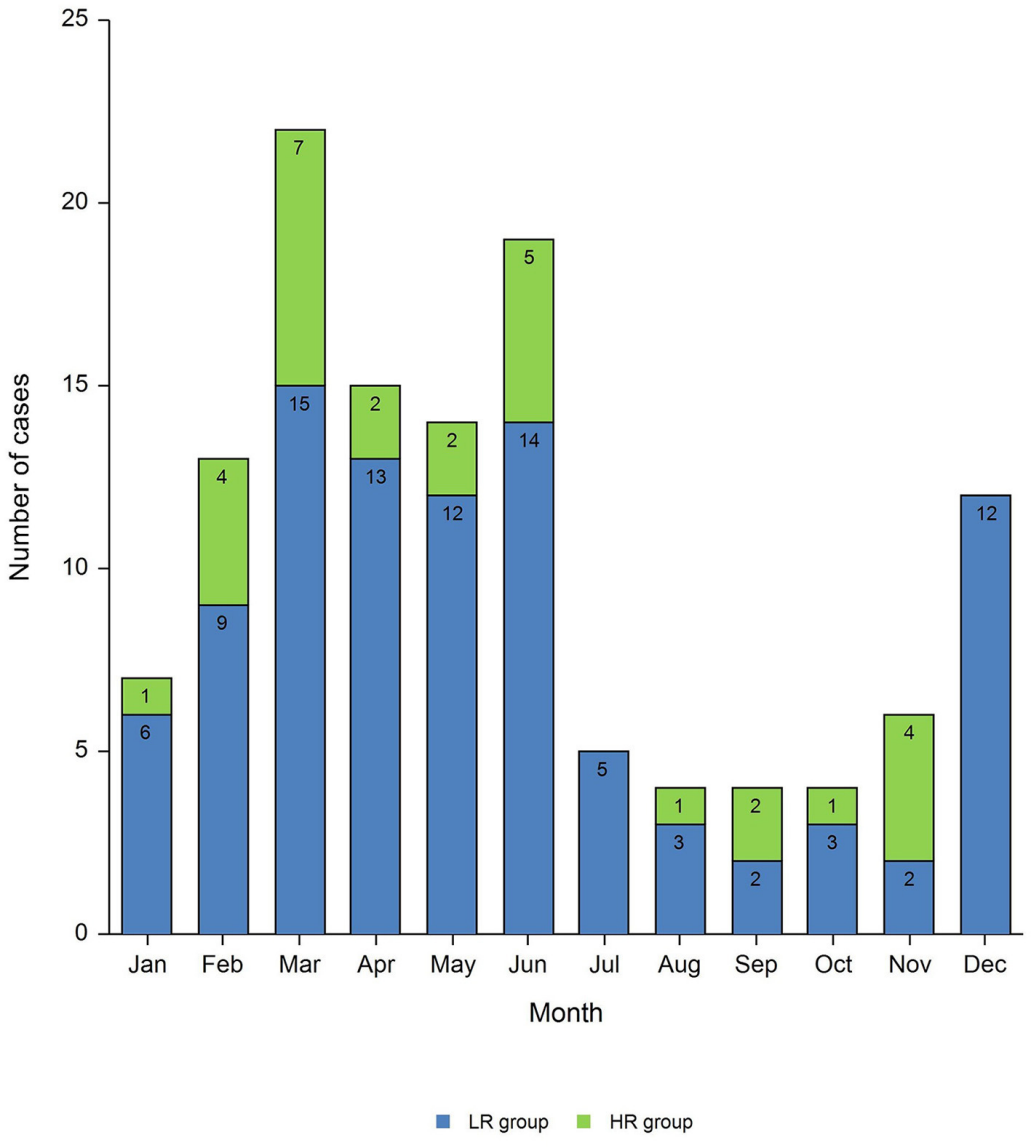
Monthly distribution of ARF diagnoses.

Most of the cases were diagnosed during spring (42% of total cases) with the peak of incidence in March (18% of total cases) and a late peak in June (15% of total cases) (Figure 1). ARF cases presented with chorea showed a delayed peak in June.

### ARF Incidence and Impact of HR Criteria for ARF Diagnosis

**Figure 2:**
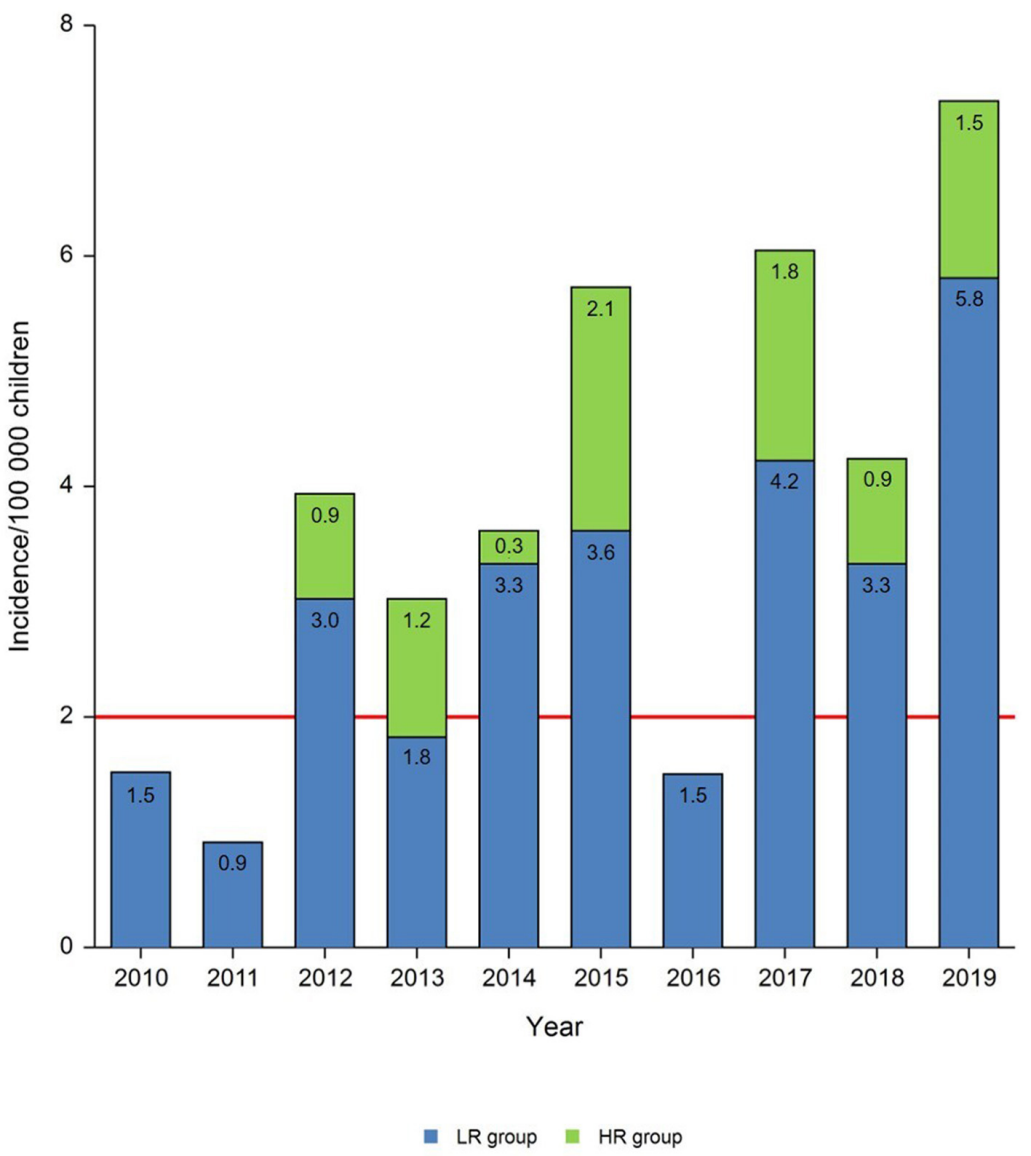
Annual incidence rates of patients with ARF per 100,000 children. The red line indicates the threshold value of incidence which differentiates low-risk from moderate and high-risk populations.

As observed in Table 2, ARF incidence was over the limit in most of the years examined, ranging from 0.91 to 7.33/100,000 cases, leading to define Tuscany an HR population area (Figure 2).

**Table 2:** ARF annual incidence rates expressed as cases per 100,000 population according to LR and HR criteria by year.

**Year**	School-age population^*^	**Incidence with LR criteria**	**Incidence with HR criteria**	**Diagnosis with LR criteria (%)**	**Diagnosis with HR criteria (%)**	**HR impact (%)**
2010	328,525	1.52	1.52	100	0	0
2011	329,234	0.91	0.91	100	0	0
2012	329,626	3.03	3.94	77	23	30
2013	330,341	1.82	3.03	60	40	67
2014	331,182	3.32	3.62	92	8	9
2015	331,848	3.62	5.73	63	37	58
2016	331,719	1.51	1.51	100	0	0
2017	331,140	4.23	6.04	70	30	43
2018	330,020	3.33	4.24	79	21	27
2019	327,293	5.81	7.33	79	21	26

* *Population at risk data derived by ISTAT census*.

The highest number of patients was registered in 2019 (*n* = 24), 19 out of them being diagnosed with LR criteria, evidencing an ARF epidemic peak.

In the years 2010 and 2011 a low incidence of ARF diagnosis with LR criteria was observed (1.52/100,000 and 0.91/100,000, respectively). Similarly, we did not find any patient who would have received diagnosis with HR criteria in the same period.

ARF incidence in 2013 (1.82/100,000) was under the limit for HR population area, but it would have increased to 3.03/100,000 if HR criteria were used.

We did not find any ARF diagnosis with HR criteria in 2016. The incidence in this year (1.51/100,000) was under the limit of HR population area, whereas both the previous and the following year were above it (5.73/100,000 and 6.04/100,000, respectively).

In the group HR patients post-2015 the number of ARF diagnoses per year varied over the years 2015 and 2019 and increased of 58 and 26%, respectively, by using diagnostic criteria for HR population. Similarly, in the HR group patients pre-2015 the use of HR criteria would have led to an increase in ARF diagnoses of 9% in 2014, 67% in 2013 and of 30% in 2012. Notably, 40% of ARF patients would have been diagnosed in 2013.

When the epidemiologic impact on total ARF diagnoses was calculated by using HR criteria, an increase of 30% was estimated (Table 2).

### Clinical Presentation: Major Criteria

Exploring major criteria in the overall cohort, joint involvement was the most common (68%) followed by carditis (58%) and Sydenham chorea (19%). Only nine patients (7%) showed erythema marginatum. No patients were found with subcutaneous nodules.

Among LR group patients, carditis was the most common criteria (72%), while 58% had polyarthritis and 46% polyarthralgia.

Among HR group patients joint involvement was always documented, the most common being the knee (27%), representing the most inclusive criterion for ARF diagnosis: 72% had monoarthritis (being the only major criterion in 17 patients), 41% polyarthralgia (being the only criterion in four). The most non-articular major criterion was carditis (14%), whereas chorea was found in only one patient (Table 3).

**Table 3:** Main clinical manifestations of patients.

	**LR Group**	**HR Group**	**Total**	***P*-value**
Carditis, *n* (%)	69 (72)	4 (14)	73 (58)	<0.001
Subclinical carditis, *n* (%)	40 (42)	3 (10)	43 (34)	0.004
Chorea, *n* (%)	23 (24)	1 (3)	24 (19)	0.01
Erythema marginatum, *n* (%)	9 (9)	0	9 (7)	0.11
Subcutaneous nodules, *n* (%)	0	0	0	–
Polyarthritis, *n* (%)	56 (58)	0	56 (45)	–
Monoarthritis, *n* (%)	0	21 (72)	21 (17)	–
Polyarthralgia, *n* (%)	44 (46)	12 (41)	56 (45)	0.83
Monoarthralgia, *n* (%)	0	0	0	–
First-degree atrioventricular block, *n* (%)	10 (10)	3 (10)	13 (10)	0.76

*–, not applicable*.

Globally, 73 out of 125 (58%) patients of the overall cohort showed a cardiac involvement which resulted significantly higher in LR group patients when compared to HR group patients, as shown in Table 3 (*P* < 0.001). Furthermore, among LR group patients, 29% presented moderate-severe mitral insufficiency. Moderate-severe aortic insufficiency was always associated with moderate-severe mitral insufficiency. Among HR group patients mild mitral valve damage was mainly recorded (75%) and no cases of aortic valve injury or combined lesions of both valves were found (Table 4).

**Table 4:** Echocardiographic findings of patients with cardiac involvement.

	**Mitral regurgitation**			**Aortic regurgitation**		
	**Mild**	**Moderate**	**Severe**	**Mild**	**Moderate**	**Severe**
LR group, *n* (%)	36 (52)	18 (26)	2 (3)	23 (33)	5 (7)	1 (1)
HR group, *n* (%)	3 (75)	1 (25)	0	0	0	0
Total, *n* (%)	39 (53)	19 (26)	2 (3)	23 (32)	5 (7)	1 (1)

Our outcomes showed a prevalence of subclinical carditis (34%) on the overall population (Table 3). Of note, the number of patients with subclinical carditis was significantly higher in LH group than in HR patients (χ^2^ = 8.4; *P* = 0.004). No patient presented valve stenosis as well as cardiovascular complications such as ventricular dysfunction at the time of diagnosis, except for one patient with mild pericardial effusion. Of note, two patients with carditis developed sequelae, one transient ischemic attack, and one dilation of the right ventricle.

Chorea showed a prevalence of 19% in our cohort and females were predominantly affected (14 out of 24). Among HR group patients chorea was not associated simultaneously with carditis, while within the LR group 22 out of 23 patients with chorea additionally presented cardiac involvement (two-sided Fisher exact test, *P* = 0.002), particularly mitral valve regurgitation.

### Clinical Presentation: Minor Criteria

Most of the patients selected in our study were admitted with fever (74%) and elevation of ESR and/or CRP (86%). No difference was found between HR and LR groups concerning the prevalence of these criteria. LR group patients showed high prevalence of polyarthralgia (46%).

Among the overall population, 10% showed prolonged PR interval for age and 10 out of 13 patients had cardiologic involvement. The median values of the PR interval among LR and HR groups were 140 ms (*n* = 10, IQR = 78) and 240 ms (*n* = 3, IQR = 120), respectively (Table 3). Moreover, other ECG abnormalities were found only in three patients of LR group: second-degree atrioventricular block (Mobitz type 1 and type 2) in two and an episode of supraventricular tachycardia in one.

## Discussion

Our results highlight that the annual incidence of ARF in Tuscany was above the cut-off (<2 out 100,000 per year), when calculated in a period of 10 years (2010-2019), with an increasing trend from 2012 to 2019. The mean incidence was over the limit for most of the years considered in the selected period. Interestingly, it remained above the threshold value either after or before the application of HR criteria leading to an increase of 30% on total ARF diagnoses. Therefore, Tuscany should be considered an HR geographic area and, according to the latest guidelines, the criteria for HR populations should be used in this region.

Investigations on the incidence of ARF along the Italian country are few and limited to the Northern Italy. Recently, Licciardi et al. (15) estimated that ARF incidence in children living in the Province of Turin (Northern Italy) in the period between the years 2007–2016 ranged from 3.8 to 12.7/100,000, when calculated using LR and HR criteria respectively. Moreover, the study concluded that the application of HR criteria led to a 20.7% increase in the ARF diagnosis. In the period between the years 2014 and 2016 the Province of Turin showed an ARF rate incidence higher than Lombardy in the Northern Italy (4.24/100,000) (16). Other investigations performed along the territory of the North of Italy were conducted in Emilia Romagna in the period between 2000 and 2014 (before the publications of the AHA guidelines), showing that the ARF annual incidence in children presented with and without carditis were 0.8–2.4/100,000 and 1.5–3.8/100,000/year, respectively (17). Recently, a retrospective analysis of the childhood population living in the province of Bologna (Emilia Romagna) in the period between 2012 and 2017 showed ARF annual incidence of above two out of 100,000 (18). The unique report about the ARF incidence in the Central Italy refers to the Breda's study which evidenced in the period 2002–2009 an ARF incidence of 4.1/100,000 (19). Comparing the above-mentioned Italian studies we observed the same overall ARF rate incidence with regional variations in the same periods analyzed. In particular, Tuscany represented the unique region in which the incidence, calculated with HR criteria, was under the limit in 2016, reaching value over the limit in the next years. This indicates that the use of HR criteria is mandatory to avoid ARF underdiagnoses. Indeed, the ARF incidence in 2017 would have changed from 6.04 to 4.23/100,000, with the loss of six cases out of 20 if the use of HR criteria had not been continued. In an attempt to explain the inter-regional differences in the ARF rate incidence, we focused on the strains of GAS-causing infections. GAS M types have been highlighted as an important determinant to clarify the distribution and development of ARF outbreaks. Genetic variation in the *emm* gene, which encodes the M protein, has been correlated with varying ARF incidence, particularly the occurrence of GAS serotype M18 isolates (20–22). The association of several outbreaks of ARF with the circulation of mucoid GAS strains in the community has been reported (23). Therefore, we might argue that the variability of ARF incidence observed in the same periods in our country could be related to the heterogeneity and resurgence of rheumatogenic strains and to the different distribution of M types, due to the mobility of GAS strains on a global scale (24).

Concerning the peak of incidence observed in our cohort in 2019, an increase in throat GAS infections untreated or not correctly treated could be the explanation. Recently Di Muzio et al. reported knowledge gaps of GAS pharyngitis diagnosis and low adherence rates to the treatment in our country, resulting in difficulties to reduce the incidence of ARF (25). This observation strongly indicates the need to improve the pediatrician's approach to diagnosis and management of GAS infection, as the decline in the incidence of ARF in developed countries in previous decades, has reduced the attention to either diagnosis or treatment of streptococcal pharyngitis.

It is known that first episodes of ARF can rarely occur in children younger than 5 years, the highest incidence being reported among children aged between 10 and 14 years, followed by those ones aged between 5 and 9 years (4, 6, 26, 27), with episodic occurrence in adolescence. Furthermore, our population showed equal distribution.

According to previous studies (15), ARF diagnosis in our cohort showed a typical seasonal distribution pattern, time between the triggered infection and the onset of clinical manifestations being after about 2–3 weeks, predominantly in March, except for chorea which developed after about 40 days from the infection, compatible with a greater latency on developing symptoms.

Arthritis was the most frequent major criterion found in our cohort. Interestingly, in LR group it presented as polyarthritis without being the more relevant, while it is always found in HR group, mainly as monoarthritis, proving to be the most inclusive sign for ARF diagnosis. This observation agrees with a previous study which underlies the clinical relevance of monoarthritis in high incidence population (28), confirming its value as major criteria only in HR population.

Clinical carditis and arthritis in our cohort showed a frequency similar to that reported in studies from the other regions of Northern Italy (15, 29).

After arthritis, carditis was the second represented Jones's criterion in our cohort. It was present in more than half of the overall cohort, significantly predominant in LR patients and mainly involving mitral valve. Similarly, subclinical carditis showed a prevalence in both groups with significant predominance in LR group. This outcome indicated a higher frequency of silent carditis (without a reduction in the frequency of total carditis) in our region than in the regions previously investigated (15, 29). Indeed, in most of the patients of our cohort the presence of polyarthralgia and monoarthritis, as first signs, oriented to a suspicious of ARF, leading to discover a silent carditis. It is known that ARF clinical features can resolve completely in weeks or months, while valvular lesions may persist and lead to RHD (30–32). The evolution of the silent carditis has not been completely clarified; however, a prospective study demonstrated that subclinical lesions can persist and sometimes even worsen (33). Since it has been observed that subclinical RHD prevalence correlates closely with ARF incidence, being considered as its surrogate measure in HR population (34), we highlight the relevance of the echocardiographic evaluation to estimate ARF incidence in HR populations.

Chorea was the third represented criterion in our cohort, females being more affected, as described in other reports (35). As opposed to our results and previous investigations (29), Pastore et al. (30) reported a higher prevalence of chorea (54%). Interestingly, we enlightened a higher frequency of carditis among patients with chorea either at presentation or during the follow-up; of note, chorea was usually associated with mild cardiac involvement, confirming that patients with chorea hardly develop severe carditis (36, 37). Therefore, we empathize that echocardiographic assessment should be routinely performed in each patient with suspected or confirmed chorea in order to identify the presence of valvulitis (even subclinical) at the time of diagnosis or developing later.

Among minor criteria, the prolongation of PR interval [occurring in over 2% of the normal population (38)] was detected in a limited number of our cases without a positive correlation with carditis or with other major criteria.

The present study has some limitations because of its retrospective design and a prospective study will be necessary to confirm our findings. Due to the lack of nationwide epidemiological data, especially in the unexplored South of Italy, an Italian registry of all cases of ARF may be a relevant aim. Finally, we point out the need of a critical approach in the application of the new ARF criteria in children presenting with fever, polyarthralgia and increase of ESR and/or CRP, particularly in HR population.

## Conclusion

The epidemiological impact of HR criteria is relevant in Tuscany, where their application led to an increase in ARF diagnoses of 30%. Thus, Tuscany should be considered an HR area and the new criteria for HR populations should be used in this region. Moreover, if the observed trend were confirmed, it would represent an inversion with respect to the past century, when a decline in the incidence of ARF in developed countries has been described as consequence of improved public hygiene and widespread use of antibiotics.

## Data Availability Statement

The datasets generated for this study are available on request to the corresponding author on reasonable request.

## Author Contributions

AMQA conceptualized and designed the study, designed the data collection instruments, contributed to collect the clinical data, conducted the final database, and drafted the manuscript. FP conducted the final database, performed the statistical analyses, and drafted the initial manuscript. ADG designed the data collection instruments, performed the statistical analyses, contributed to interpretation of data, and drafted the initial manuscript. SC, GB, SA-R, AB, and AO contributed to collect the clinical data, carried out the initial analyses, and critically reviewed the manuscript for important intellectual content. DP, NA, CG, and GS conceptualized and designed the study, supervised data collection, contributed to interpretation of data, and critically reviewed the manuscript for important intellectual content. RC conceptualized and designed the study, coordinated and supervised data collection, contributed to interpretation of data, and critically reviewed the manuscript for important intellectual content. All authors approved the final manuscript as submitted and agree to be accountable for all aspects of the work.

## Conflict of Interest

The authors declare that the research was conducted in the absence of any commercial or financial relationships that could be construed as a potential conflict of interest.

## Publisher's Note

All claims expressed in this article are solely those of the authors and do not necessarily represent those of their affiliated organizations, or those of the publisher, the editors and the reviewers. Any product that may be evaluated in this article, or claim that may be made by its manufacturer, is not guaranteed or endorsed by the publisher.

## Abbreviations

Anti-DNase B: Anti-Deoxyribonuclease B
ARF: Acute Rheumatic Fever
ASO: Antistreptolysin-O
CRP: C-reactive protein
ECG: Electrocardiography
ESR: Erythrocyte sedimentation rate
GAS: Group A Streptococcus
HR: High-Risk
LR: Low-Risk
RHD: Rheumatic Heart Disease.
